# National Association of Dental Examiners

**Published:** 1889-10

**Authors:** 


### National Association of Dental Examiners.

The National Association of Dental Examiners held its eighth annual session in the Town Hall, Saratoga Springs, N. Y., commencing Tuesday, August 6, 1889, at 1 o'clock P. M.

The following State Boards of Examiners were represented: Illinois by C. R. E. Koch; Ohio, J. Taft, H. A. Smith; New Jersey, F. A. Levy, J. G. Palmer; Indiana, S. T. Kirk; Maryland, T. S. Waters; Massachusetts, L. D. Shepard, J. S. Hurlbut; Vermont, Geo. H. Swift, James Lewis; Delaware, C. R. Jefferis, T. H. Gilpin; Colorado, P. T. Smith; Georgia, A. G. Bouton.

Delaware and California were admitted to membership.

Drs. Jefferis, Shepard, and Koch were appointed a committee to consider a mass of correspondence with reference to the standing of a college whose name had been omitted from the list of colleges whose diplomas were recommended to be received by the State boards. This committee was afterwards constituted the Committee on Colleges.

The committee at a later session reported, recommending that the secretary be instructed to inform the Dental Department of St. Louis College of Pysicians and Surgeons that owing to insufficient information the association is unable to take final action on its application for recognition; and sustaining the action of the officers in omitting the name of the University of Maryland, Dental Department, from the printed list of recognized colleges last year. The report was received and adopted unanimously.

The committee also reported the following list of colleges which may be recommended as reputable by this association:

American College of Dental Surgery, Chicago, Ill.

Baltimore College of Dental Surgery, Baltimore, Md.

Boston Dental College, Boston, Mass.

Chicago College of Dental Surgery, Chicago, Ill.

College of Dentistry, Department of Medicine, University of Minnesota, Minneapolis, Minn.

Dental Department, Columbian University, Washington, D. C.

Dental Department of Northwestern University, Chicago, Ill.

Dental Department of Southern Medical College, Atlanta, Ga.

Dental Deparment of University of Tennessee, Nashville, Tenn.

Harvard Unversity, Dental Department, Cambridge, Mass.

Indiana Dental College, Indianapolis, Ind.

Kansas City Dental College, Kansas City, Mo.

Louisville College of Dentistry, Louisville, Ky.

Minnesota Hospital College, Dental Department, Minneapolis, Minn. (Defunct.)

Missouri Dental College, St. Louis, Mo.

New York College of Dentistry, New York City.

Ohio College of Dental Surgery, Cincinnati, O.

Pennsylvania College of Dental Surgery, Philadelphia, Pa.

Philadelphia Dental College, Philadelphia, Pa.

School of Dentistry of Meharry Medical Department of Central Tennessee College, Nashville, Tenn.

St. Paul Medical College, Dental Department, St. Paul Minn. (Defunct.)

University of California, Dental Department, San Froncisco, California.

Northwestern College of Dental Surgery, Chicago, Ill. (Defunct.)

University of Iowa, Dental Department, Iowa City, Ia.

University of Maryland, Dental Department, Baltimore, Md.

University of Michigan, Dental Department, Ann Arbor, Mich.

University of Pennsylvania, Dental Department, Philadelphia, Pa.

Vanderbilt University, Dental Depaıtment, Nashville, Tenn.

The committee recommended also that the standing Committee on Colleges be instructed hereafter to take cognizance of and investigate all charges against any college, that they give the accused an opportunity for defense, and that they report a revised list of colleges at each annual meeting after having investigated all complaints; and that this same committee also have authority to inquire into the proper equipment and organization of colleges not now on our list, so that they may be able to report as to the capability of such institutions to give acceptable instruction, both as to the quality and quantity of its teaching.

After hearing the representative of the College of Dental Surgery of the University of Denver, Dr. P. T. Smith, that institution was added to the list, and the report was then adopted.

Dr. Koch offered resolutions that it is the sense of this association that no one should be permitted to assume the responsibilities of a dental practitioner until he shall have had at least three years' previous study and instruction, inclusive of three full terms of not less than five months each, in a properly organized and equipped dental college, provided that time spent in the study of medicine or graduation from a medical college may be credited on this requirement not to exceed the period of two years, or two full terms of collegiate instruction; and recommending to such State boards of dental examiners as are by the laws of their respective States required to issue licenses to practice dentistry to all holders of diplomas from reputable dental colleges, that they make such rules as shall require all colleges to make three full calendar years of study, and the attendance upon three full college terms, of not less than five months each, a prerequisite to graduation; and, that only such colleges as shall comply with this rule on or before the beginning of their scholastic year of 1890–91, should thereafter be considered as reputable; and that all State boards should, when their State laws permit the same, decline to grant a license to practice to any one who can not produce evidence showing that he has spent at least three full years in study and preparation, before attempting to assume the responsibilities of a dental practitioner.

Referred to a committee consisting of Drs. Kirk, Palmer, and Bouton, with the information that the National Association of Dental Faculties had adopted a rule to go into effect at the session of 1891–92 requiring attendance upon three full regular courses before examination for graduation. The committee reported recommending that the portion "relating to States where an examination is held and license granted be approved." The report was adopted.

Dr. Koch moved that the Secretary be instructed to publish a notice in the dental journals informing all dental colleges not now recognized as reputable by this association that in order to be enrolled upon the list of colleges recognized by it, it will be necessary for such colleges to apply for recognition and show that their workings are such as to entitle them thereto. So ordered.

Dr. Shepard moved to make the standing Committee on Colleges consist of five members, whose duty it shall be to report annually upon the colleges entitled to recognition. So ordered.

The following officers were elected: T. S. Waters, Baltimore, President; C. R. E. Koch, Chicago, Vice-President; F. A. Levy, Orange, N. J., Secretary and Treasurer. The President appointed as the Committee on List of Reputable Dental Colleges, Drs. L. D. Shepard, C. R. E. Koch, C. R. Jefferis, F. A. Levy, and S. T. Kirk.

Adjourned to meet at the time and place of the next meeting of the American Dental Association, at 9 A. M. of the first day.

---

EXCESSIVE PERSPIRATION.—A circular has been sent to all the medical officers in the Prussian army, recommending chromic acid to check excessive perspiration; a 10 per cent. solution, applied to the feet every three or six weeks, completely controls the trouble.

---

UNDER the will of the late Dr. Alonzo Clark a scholarship was endowed at the College of Physicians and Surgeons, New York, for the promotion of the discovery of new facts in medical science. This scholarship has an annual income of about nine hundred dollars, and the faculty of the college have worthily bestowed it for a term of three years upon Dr. T. Mitchell Prudden.—*Medical News*, August 10, 1889.

